# The taming of the neural crest: a developmental perspective on the origins of morphological covariation in domesticated mammals

**DOI:** 10.1098/rsos.160107

**Published:** 2016-06-01

**Authors:** Marcelo R. Sánchez-Villagra, Madeleine Geiger, Richard A. Schneider

**Affiliations:** 1 Palaeontological Institute and Museum, University of Zurich, Karl-Schmid-Street 4, 8006 Zurich, Switzerland; 2 Department of Orthopaedic Surgery, University of Californiaat San Francisco, 513 Parnassus Avenue, S-1161, San Francisco, CA, USA

**Keywords:** ontogeny, modularity, dog, pleiotropy, island, evolutionary developmental biology

## Abstract

Studies on domestication are blooming, but the developmental bases for the generation of domestication traits and breed diversity remain largely unexplored. Some phenotypic patterns of human neurocristopathies are suggestive of those reported for domesticated mammals and disrupting neural crest developmental programmes have been argued to be the source of traits deemed the ‘domestication syndrome’. These character changes span multiple organ systems and morphological structures. But an in-depth examination within the phylogenetic framework of mammals including domesticated forms reveals that the distribution of such traits is not universal, with canids being the only group showing a large set of predicted features. Modularity of traits tied to phylogeny characterizes domesticated mammals: through selective breeding, individual behavioural and morphological traits can be reordered, truncated, augmented or deleted. Similarly, mammalian evolution on islands has resulted in suites of phenotypic changes like those of some domesticated forms. Many domesticated mammals can serve as valuable models for conducting comparative studies on the evolutionary developmental biology of the neural crest, given that series of their embryos are readily available and that their phylogenetic histories and genomes are well characterized.

While the evolutionary origin of neural crest has attracted much attention, its subsequent evolution has received almost no attention and yet it is more readily open to experimental investigation and has greater relevance to understanding vertebrate evolution.

—Donoghue *et al.* [1, p. 530]

## 1. Introduction

New appraisals of molecular and archaeological data are illuminating the origins of domestication [2,3] and genomic data are providing insights into diverse subjects [4], including the relationships among wild progenitors and subsequent breeds [5–16] and the mechanisms for adaptations to different diets and locomotory patterns [17,18]. Domesticated forms are experiments in evolution, as selective breeding has produced rapid phenotypic changes that otherwise would occur in geological time. The interest in domestication has not waned since Darwin (1868) devoted so much effort to the subject [19–22]; this is true for plants and animals, the latter the subject of this review. But a developmental perspective has largely been lacking, in spite of its centrality to current evolutionary theory [23]. Information from development is necessary to understand the disparity among breeds [24–27] and the basis for domestication in different species [28]. For this reason, the provocative hypothesis most recently articulated by Wilkins *et al.* [29] on a potential common developmental mechanism underlying all domestication in mammals deserves a closer look and a critical discussion.

**Figure 1.**
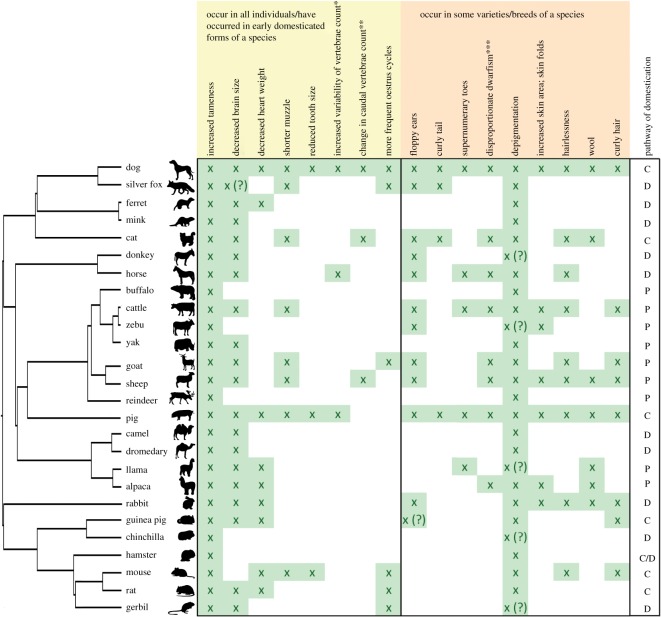
Occurrence of features of the ‘domestication syndrome’ in domesticated mammals ([29,30] and references therein) [33–36] and the hypothesized mode of domestication for them [2,31]. The mode of domestication can be of different kinds: along the (i) ‘commensal’ pathway, animals are attracted to and taking advantage of elements of the human niche and subsequently develop social and/or economic bonds with humans. The ‘prey’ and ‘directed’ pathways, on the other hand, are initiated by humans. The species undergoing the (ii) ‘prey’ pathway are usually prey species which are domesticated following continuous stages of game management strategies, herd management strategies, and controlled breeding. The (iii) ‘directed’ pathway is an immediate and fast way of domestication, using established knowledge about previous domestication processes (reviewed in [37]). We refer here to traits hypothesized to have been fixed in the initial process of domestication, and not to ‘improvement traits’ [2,38] present only in a proportion of domesticates. The length of the branches is proportional to time of divergence, based on conservative estimates for the divergence among species from different sources [39–43]. A test for the presence of a phylogenetic signal [44,45] for each feature was performed using the Mesquite software [46]. Of the characters hypothesized to have occurred in early domesticated forms, only ‘more frequent oestrus cycles’ shows phylogenetic signal which is statistically significant. C, commensal; D, directed; P, prey pathways; asterisks indicate: ‘*’, thoracic, lumbar; ‘**’, increase or decrease; ‘***’, relatively short limbs.

Domestication has led to increased phenotypic variation and phenotypic novelty not observed in wild forebears [30]. Despite the different paths that may lead to domestication ([31,32]; figure 1), the occurrence of phenotypic alterations associated with domestication in animals is often similar in diverse and unrelated groups [21]. In mammals, this has been called the ‘domestication syndrome’ [31,47], although the concept of a ‘domestication syndrome’ has also long been widely used to describe a similar phenomenon in crops and other cultivated plants [48–51].

Because the term ‘syndrome’ in this context does not refer to a specific pathological condition, one might prefer the use of a word such as ‘complex’. But the concept of characterizing this type of evolutionary phenomenon as a ‘syndrome’ has a long history in the literature, especially concerning the domestication of plants, where humans have selected ‘for interrelated syndromes of characteristics’ [52, p. 314]. Similarly, Faegri & Van der Pijl [53, p. 23], when explaining the coevolution of various parts within a blossom in relation to pollination mechanisms, stated that ‘the constant occurrence together in nature shows that the combinations of characters involved in a syndrome are far from being accidental or redundant’. Thus, we will follow the convention of using the term ‘syndrome’ despite its somewhat negative connotation in the context of neurocristopathies.

Among other characters, the following features are associated with domestication in mammals: increased docility, increased skillfulness in using human cues (gestures and glances), increased fecundity (including non-seasonality of oestrus cycles, hormonal changes, multiple breeding cycles per year and earlier sexual maturity), reduction of tooth size, shortening of the rostrum, reduction of brain size, floppiness of the ears, curliness of the tail and depigmentation of skin and fur [30]. The coupling of characters indeed was at the core interest in the famous and on-going studies initiated in 1959 by D. K. Belyaev in Novosibirik, Siberia. In breeding experiments on silver foxes (a colour phase of the red fox), mink and rats, Belyaev showed that selection for specific behaviours such as tameness leads to the expression of characteristics typical of the ‘domestication syndrome’ [33,54–58]. Already, after relatively few generations, the foxes were increasingly tame and docile, expressed aberrant pigmentation, floppy ears, rolled and shortened tails, shorter limbs, shortened and widened rostra, smaller brains, earlier sexual maturity and ability to perceive human gestures.

Some authors have pointed out the potential connection of these features to neural crest development and that neural crest cells have served as a conduit for the simultaneous evolution of multiple phenotypic traits [59–62]. Crockford [60,63] and Wilkins *et al.* [29] discussed this subject explicitly and in detail, so much so that the ‘domestication syndrome’ in mammals is now being referenced as indicative of the presence of the ‘syndrome’ and neural crest involvement [13,29]. According to Wilkins *et al.* [29], the selection for tameness leads to mild neural crest cell developmental deficits during embryonic development, which either directly or indirectly, cause most the characteristics of the ‘domestication syndrome.’

We recognize two fundamental aspects in the ideas on the domestication syndrome: (i) the frequency and covariation of the traits and (ii) the role of the neural crest. These deserve critical treatment as they concern the developmental morphology of animals that interact very closely with humans, and they illustrate the generative and regulatory role of development in evolution (*sensu* Alberch [64]).

## 2. On the occurrence of morphological features in domesticated forms

Not all domesticated mammals present the totality of features predicted by the ‘domestication syndrome’ and in most cases, just a subset of them occurs (figure 1). The full ‘syndrome’ is found in dogs and includes remarkable variation in features such as rostrum length (i.e. jaw size), coat colour and behavioural sequences [54,58,65–67]. Interestingly, another group with a somewhat similar pattern are foxes, also a canid—underscoring the potential relevance of considering phylogeny [39,68] when searching for mechanisms underlying the genetic integration of developmental programmes or modules. However, the frequency and covariation of the non-variant main features in the domestication syndrome are not significantly tied to phylogeny in six out of seven cases (figure 1).

We have compiled the information on the distribution of features to the best of our knowledge, based on a critical and exhaustive review of the literature. However, clearly some patterns may warrant revision, as knowledge of the wild populations for many species is either deficient or at best only indirectly available. The silver fox is a good example. Although this case has been well documented and is broadly cited, having greatly influenced ideas on the ‘domestication syndrome’ and its bases [29], the experiment was conducted on farmed (not-wild) foxes (D. Kruska 2016, personal communication; [55]). The extent to which this fact has affected this classic and important experiment as a model for domestication has to our knowledge never been explored.

There are many differences in the modes and extent of selective breeding in the history of the species depicted in figure 1. One of the differences is the antiquity of the domestication process. Here, we purposely avoid the term ‘event’, as the integration of genomic and archaeological data is demonstrating the complexity of the history of domestication for each species, with potentially multiple and parallel ‘events’ and population admixtures [69]. Ongoing work is revising and refining the estimates of the beginnings of domestication for mammalian species [2,70].

## 3. On the potential role of neural crest in the concerted occurrence of ‘domestication syndrome’ features

Wilkins *et al.* [29] suggested that a ‘mild neurocristopathy’ leads, as a by-product, to all the observed components of the ‘domestication syndrome’. Neurocristopathies are complex and often severely pathological syndromes that span multiple organ systems and morphological structures and are united by abnormal migration, differentiation, division and/or survival of neural crest cells [71,72]. Neural crest cells emerge from the dorsal margins of the neural tube during early development and migrate along stereotypical pathways to a number of sites. They differentiate into a wide range of ectomesenchymal (e.g. bone, cartilage and dentine) and non-ectomesenchymal (e.g. neurons, glia, pericytes and melanocytes) derivatives [1,73,74]. The neural crest is the source of secretory cells and connective tissues in glands such as the adrenal that produces epinephrine, norepinephrine and dopamine, and in the pituitary, thymus, thyroid and parathyroids [60,75–79]. Neural crest cells are also the source of the pigmented dopaminergic neurons in the substantia nigra, a brain region associated with learning and reward [80,81]. Neural crest-derived melanocytes are the source of pigmentation throughout the head and body, and neural crest-derived dermis in the head provides species-specific pattern to the integument and its various appendages, such as hair, feathers, beaks and horns [61,82]. All the bones, cartilages and muscle connective tissues (e.g. tendons) in the head originate from neural crest cells. Given the broad range of neural crest derivatives across multiple systems, regulatory changes to the neural crest can be a major source of evolutionary transformations in behaviour, the integument and the skeleton [61].

What predictions does the neural crest hypothesis of the ‘domestication syndrome’ make about the development of this population of progenitor cells? To answer this, we need to consider the embryological parameters that could be agents of change in neural crest evolution. These include: (i) timing of emigration, (ii) overall size of the progenitor pool, (iii) allocation and/or regional distribution of sub-populations (e.g. midbrain versus hindbrain), (iv) specification of lineages (e.g. cell types and derivatives), (v) growth parameters (i.e. proliferation rates and timing of differentiation), (vi) signalling interactions with adjacent and non-neural crest-derived tissues, and (vii) regulatory changes affecting spatial and temporal domains, and levels of gene expression. Details on these parameters for different species of vertebrates are very limited, and this much needed fundamental and comparative work is especially lacking for mammals.

The relative timing of neural crest development to other events and its relation to adult anatomy has been studied in only a handful of species. In frogs, the relation is far from straightforward. Mitgutsch *et al.* [83, p. 255] studied the development of the cranial neural crest, neural tube differentiation and somite formation in discoglossid frogs and found that they ‘…could not identify any obvious relation of our embryonic data with peculiarities of post-embryonic stages. Cell populations contributing to head mesenchyme interplay in a highly integrated yet developmentally plastic manner’. On the other hand, the comparison of marsupials and placentals within mammals suggested a coupling of relative timing of neural crest cell migration and development of adult structures: cranial neural crest in *Monodelphis domestica* develops relatively early compared with other embryogenesis events among the placentals investigated to date: mice, rats and macaque monkeys [84–86]. Neural crest-derived oral and facial structures develop very early in marsupials, in close association with their lactation after a short gestation.

More than any other experimental model systems, those involving birds have provided critical insights into how changes to the neural crest during development can affect morphological evolution. Studies in birds have illuminated key morphogenetic events that probably generate phenotypic variation in many of the traits associated with the domestication syndrome, especially in the craniofacial complex and the integument. In particular, the use of a quail–duck chimeric system [59,87–89] has revealed how a series of developmental mechanisms control the size of the neural crest-derived jaw skeleton during three main phases of embryogenesis [90,91]. First, when the anterior neural tube becomes subdivided, ducks have a much broader midbrain from which the neural crest-derived jaw progenitors migrate, and this provides them with a 15% larger initial pool of cells to generate the jaw skeleton [92]. Second, when this population of jaw progenitors expands, there is species-specific control over the cell cycle, which is neural crest-mediated and quickly doubles the size of the duck jaw primordia relative to stage-matched quail. Neural crest cells accomplish this task by differentially regulating and responding to various signalling pathways that are known to affect proliferation and exit from the cell cycle, and by executing autonomous molecular and cellular programmes for cartilage and bone that are intrinsic to each species [59,89,93–98]. For example, by the time the jaw skeleton becomes mineralized in quail, expression levels for the transcription factor *Runx2* are more than double those of ducks [96]. Experimentally increasing levels of *Runx2* in chick embryos markedly decreases the size of the beak skeleton [96,99], which supports the postulated relationship between *Runx2* tandem repeat length and facial length reported for adult dogs ([100–102], but see Pointer *et al.* [102] for mammals). Thus, another mechanism that affects jaw length is the way neural crest cells establish tight control over both the expression levels of key transcription factors and the timing of skeletal differentiation [90,91].

In addition to controlling the deposition of bone and cartilage, neural crest cells also execute autonomous molecular and cellular programmes for matrix resorption through patterns and processes that are intrinsic to each species. In fact, the amount of bone resorption in quail and duck embryos is inversely proportional to jaw length, bone resorption is neural crest-mediated and modulating bone resorption can lengthen or shorten the jaw skeleton [90]. Overall, the special ability of neural crest cells to maintain spatio-temporal control over the induction, differentiation, deposition, mineralization and also the resorption of skeletal tissues is what links the determinants of craniofacial size and shape across multiple embryonic stages and is what gives neural crest its unique potential to generate craniofacial variation throughout development and evolution.

## 4. On the segregation of features within species: the case of variation across breeds in dogs

Dogs are well studied and canids are at the centre of the neural crest hypothesis, so a focus on their behaviour and morphology is particularly relevant to discuss ions on the domestication syndrome. We consider here the fact, to paraphrase George Orwell, ‘all dogs are tame, but some dogs are more tame than others’, and examine the coupling or segregation of traits. In dogs, the occurrence of features of the domestication syndrome cannot be related to different degrees of tameness among the breeds, as shown in the examples as follows.

Brachycephalic breeds, those with a relatively short and broad rostrum and broad skull, have been argued to be less fearful towards strangers than breeds with relatively longer rostra [103]. Assuming a coupling of the neural crest to features of the domestication syndrome, a short face might be expected to be linked to increased tameness and docility. However, the short rostrum of brachycephalic dog breeds is a result of secondary breeding efforts, not associated with the changes during initial domestication, and thus not related to the domestication syndrome [25,29,54,58]. Solid colour English cocker spaniels are significantly more likely to exhibit aggressive behaviour towards humans than parti-colour breed members [104], but there is likely no causal link between coat colour and aggressiveness [104].

Domestic dog breeds that are characterized as especially dominant over the owner and snapping at children (e.g. miniature schnauzer, chow chow and Scottish terrier) and breeds that are not dominant over the owner and do not snap at children (e.g. golden retriever, collie and bloodhound) exhibit a mixture of traits that are associated with the domestication syndrome and no clear association of temperament and appearance in the sense of the domestic syndrome can be found [105]. In many countries, domestic dogs are categorized according to their potential of danger to humans owing to their potential breed-specific aggressiveness. In the canton of Zurich, Switzerland, for example, some breeds have been classified as constituting an increased potential of danger, and thus, importing, breeding or keeping them is prohibited. These are American Staffordshire terrier (pit bull terrier), Staffordshire bull terrier, (American) bull terrier, American pit bull terrier and crosses thereof (e.g. Bandong). According to the breeding standards of the Fédération Cynologique Internationale, the Staffordshire bull terrier has been described to exhibit a short rostrum, half pricked ears (erect ear that slightly folds over at the tip) or rose ears (backwards drooping ear), and a wide variety of coat colours, including white. A similar appearance is also found in the American Staffordshire terrier. If mild neurocristophathy would be the major driver of the characteristics of the domestication syndrome, and no segregation of features would occur, one would not expect to observe a shortened rostrum, non-erect ears and depigmentation of fur in these, apparently, aggressive breeds.

However, segregation of behavioural traits among breeds associated with phenotypical peculiarities that fit the neural crest hypothesis of the domestication syndrome can be found among working dogs. Coppinger *et al.* [54,65] suggested that herding dogs (breeds developed to herd livestock, e.g. border collie) have been selected for a characteristic behavioural pattern which is different from livestock guarding dogs (livestock protecting dogs, e.g. Maremma). Herding dogs retain certain segments of the predatory sequence, which in wild canids can be described as ‘orient—eye—stalk—chase—grab-bite—crush-bite—eating behaviour’. Livestock guarding dogs, on the other hand, generally do not exhibit even one component of this predatory sequence but rather display social play behaviour, which is a component of the juvenile behavioural repertoire that precedes the onset of the adult predatory sequence. Livestock guarding dogs can thus be described as behaviourally juvenilized concerning predatory behaviour. Interestingly, this behavioural pattern is, at least partially, mirrored in the outer appearance of these dogs: herding dogs often exhibit erect ears and a relatively long and narrow head, whereas livestock guarding dogs often have a relatively short and broad head with a pronounced stop and floppy ears; traits which are characteristic for neonates of wild canids. In a study on neurochemical and behavioural correlates in the same groups of working dogs, low levels of predatory behaviour were correlated with low neurotransmitter levels (dopamine and norepinephrine) in herding dogs (i.e. border collie) and livestock guarding dogs (i.e. Shar Planinetz) [80]. While regulatory changes within the neural crest-derived neurosecretory cells of the neuroendocrine system could affect neurotransmitter levels and breed-specific behaviours, the underlying molecular, developmental and genetic mechanisms remain obscure.

While an argument can be made that all dogs regardless of breed are more tame than their wild ancestors, the lack of clear associations among morphological traits and degrees of tameness among breeds or strains of domestic dogs exemplifies the complexity of the genetic mechanisms underlying the generation of the domestication syndrome and a decoupling of its single features from one another in the course of domestication and artificial selection and breeding. Such uncoupling is best exemplified by the radical work of Stockard [106], which reveals the dire morphological consequences of crossing breeds that are extremely disparate in size and shape.

Similarly, experiments in other model organisms can provide insights into the segregation of traits. Two lines of rats (*Rattus norvegicus*) which have been selected from more than 60 generations for increased tameness or aggression towards humans were intercrossed and quantitative trait loci (QTL) for tameness were identified [107]. Additional QTL for white colour patches of the fur were detected, but they showed no linkage to tameness. Therefore, the authors found no evidence for white patches of fur being caused by the same loci that contribute to tameness at least in these strains. White spotting in dogs is caused by mutations in MITF, a transcription factor with a critical role in several cell types originating from the neural crest [108] but no link between coat colour and behaviour has yet been established [109]. Moreover, in the growing literature on the genomics of domestication, there has yet to emerge compelling evidence that any component of the domestication syndrome is a by-product of selection for tameness. In fact, a recent and comprehensive study demonstrated a highly polygenic basis for tameness in rabbits [110].

## 5. Single traits of the domestication syndrome

Some of the features of the domestication syndrome deserve further investigation as little is known about them. Detailed anatomical surveys of domesticated forms remain incomplete and are still needed to evaluate fully the developmental integration resulting from domestication. Even classically studied features, such as teeth, have received a thorough quantification of the effects of domestication on shape only in pigs [111]; examinations on changes in tooth size exist for dogs [24,112] and the frequency of anomalies in numbers and crowding of teeth has been documented for few species [30]. Other features are also minimally characterized. There are derivatives of the neural crest cells, such as in the gut, which have not been systematically evaluated in domesticated forms as compared with wild counterparts. The same is true of many specific structures, such as the footplate of the stapes [113], which can greatly vary in shape among wild species [114].

Recent work has shown that the pinna in mice is derived at least partially from neural crest cells [113,115]. Given that the origins of drooping ears are not entirely understood, comparative anatomical studies of the cartilage of the pinna in domestic mammals would be worthwhile. Whether a deficiency of the cartilage or an increase in relative size of the pinna leads to a non-erect state of the external ear remains unclear.

The proximate causes of curly tails are not well investigated either and how this trait relates to anatomical peculiarities of domestic animals and also to neural crest cell development, if at all remains unclear. This, like others, may represent structures not derived from the neural crest, but ultimately altered by neural crest-mediated endocrine effects on morphology [106].

## 6. On domestication and heterochrony

Heterochrony, in particular, paedomorphosis has been suggested to underlie certain behavioural and morphological changes during domestication. The case of silver foxes has been explicitly discussed in this context: the features are reportedly increased docility, depigmentation, floppy ears, curly tails and shortening and widening of the facial part of the skull [33,55,57]. First, the exploratory activity and fear response in silver fox pups selected for tameness lasts longer than in foxes from control strains. Second, melanoblast migration into depigmented areas is delayed in foxes selected for tameness—although to our knowledge no genetic evidence for co-segregation has been provided. Third, apparently, the tail is curled and the ears droop in cubs [57]. Fourth, the alterations in skull shape in some foxes of the tame strain mirror the morphological changes in early domestic dogs, which were characterized by relatively short and broad rostra. This peculiarity has repeatedly been hypothesized to be the result of a juvenilization associated with dog domestication [24,54,112,116–120]. As far as the skull is concerned though, work in domestic dogs has repeatedly shown that the short and wide skulls in certain breeds are not the result of global neotenic (paedomorphic) growth but are instead neomorphic, novel features [24,25,54,58,121,122].

While some morphological traits seem to be neotenic, reproductive traits are not. Most domesticated mammals, including the foxes which have been selected for tameability, exhibit a non-seasonal reproduction pattern, a bi-annual oestrus cycle, and reach sexual maturity earlier [30,33,55,57]. As opposed to the morphological traits, these changes are apparently more strongly affected by social and environmental factors, at least in domestic dogs [58]. Overall, the ‘domestication syndrome’ could appear to imply that domesticated forms are paedomorphic, looking like more juvenilized versions of their ancestors. This may be true for some morphological features, but not for all. Furthermore, many life-history traits do not follow the same pattern of timing change (absolute or relative).

## 7. Parallels between domestication and island evolution: an ‘island syndrome’?

**Figure 2.**
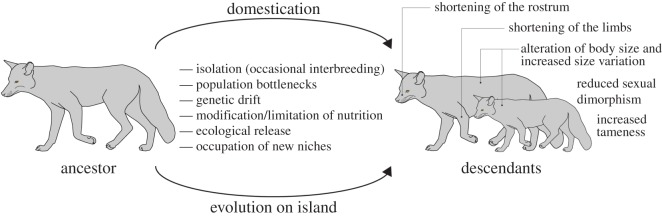
Many characteristics of domesticated mammals can also be frequently observed in mammals which evolve, or have evolved, on islands.

Many island mammals [123] exhibit phenotypic features also found in several domesticated mammals (figure 2), as in the alteration of body size, shortening of the rostrum and of the limbs, reduced sexual dimorphism [124] and reduction of brain size in some species, as well as tameness. In both domestication and in island evolution, morphological changes tend to occur relatively fast [125,126]; there are population bottlenecks [19,123,127–129], genetic drift [19,123], the occupation of new niches [30,123,130] and altered selection pressures. While on some islands there can be total isolation from the ancestral population [123], in other cases, post-divergence gene flow between wild and domestic populations is common [69], as reported for dogs [16,127,131], pigs [132], cattle [133] and horses [134]. Selective breeding (i.e. artificial selection) occurs under domestication, whereas in island environments populations are exposed to novel selection regimes. In islands, there can be a modification/limitation of nutrition and ecological release, in some cases owing to decreased interspecific competition and/or absence of predators [123,135–139], which can lead to tameness.

The similarity of domestication and island evolution phenotypic patterns has resulted in extinct island forms being interpreted as the result of domestication. This is the case of the Balearian ‘mouse goat’ *Myotragus balearicus* [19,140] and the Falkland island fox *Dusicyon australis* [141]. The Falkland island fox has been considered a feral domestic canid because of its white tail tip, rostrum and lower limbs, broad skull, bulbous forehead and its tameness [141]. This hypothesis has been rejected on the basis of divergence times, which are too old as to support the hypothesis of human transportation of these animals to the island [142,143]. *Myotragus balearicus* is well studied and it is clear that island evolution and not domestication is the reason for its phenotypic and life-history features [140,144].

The differences in the concert of factors associated with the novel environmental conditions and associated selection pressures, e.g. ecological release, under domestication and in island environments are probably crucial for the occurrence of the differences among species, as well as the phylogenetic inertia characteristic of each clade involved [137,145–147]. The similarity of some patterns does not necessarily imply similar mechanisms. For example, the decrease in brain size in island mammals is associated with a reduction in body size, whereas this need not be the case in domesticated mammals [148,149]. Examining the distribution of features of island mammals, in a phylogenetic context, can provide insights into shared developmental biases that should also be included as mechanisms that explain such phenotypic patterns.

## 8. Conclusion

The features of the ‘domestication syndrome’ are not universal among domesticated mammals, rejecting a simple and single explanation for the phenotypic patterns of domesticated forms. However, some patterns resemble the aetiology of syndromes that span multiple organ systems and morphological structures in humans and other mammals: neurocristopathies, related to the neural crest.

Changes to neural crest development may simultaneously be a major source of evolutionary variation in behaviour, the integument and the facial skeleton, with the initial selection for tameness leading to a modulation of neural crest input into the sympathetic and adrenal systems. Modifications in the activity of tyrosine pathway enzymes may enable changes in the phenotypic integration of epidermal, nervous and endocrine tissues. But this is only the start of an idea. The lack of a universal pattern begs the question of how features can become segregated, and how plasticity and modularity shape the transformations observed during domestication and the origin of the remarkable diversity among breeds of almost all domesticated forms. Through hybridization and selective breeding, individual behavioural and morphological features can be reordered, truncated, augmented, uncoupled or deleted.

A search for a common genetic mechanism to explain brain evolution in the frontal cortex of three pairs of domesticated and wild species reported that the majority of gene expression changes in dogs and wolves, pigs and wild boars, and domesticated and wild rabbits are specific for each pair [150]. This seemingly contradicted the expectation of similar underlying molecular mechanisms, or rather simply showed that some developmental aspects at the cellular level resulted in these differences among species. In any event, the results emphasize the relevance of phylogeny, as in each clade the causative variants of behavioural changes associated with domestication [151] are more distinct between clades than within clades.

To study the effects of selection for tameness in morphological traits and the developmental bases of such changes, several species currently studied under laboratory conditions could serve as models, including laboratory mice among others [152]. Here the criteria on which to define tameness should be made clear. Tameness refers to the reduction of fear, for which tests can be made (e.g. a ‘novel object’ test). Non-mammalian species could also serve as models of study. A good subject could be the Bengalese finch, domesticated forms of which show features associated with the syndrome, a species that has been extensively studied neurobiologically [153]. However, the difficulty of obtaining samples of the wild form for comparison would be an issue. Another model could be found among some species of cichlid fishes which have been raised in experimental conditions and have become tamed [154].

Some morphological features of domesticated mammals, which were considered to be the result of juvenilization, have proved not to be so. This does not exclude the potential relevance of heterochrony in the evolution of domesticated forms. The neoteny hypothesis for human evolution attempted to provide a simple and universal explanation, which proved to be too simplistic to work, but nevertheless stimulated much empirical and conceptual advances in the field [155–157].

Research on domesticated animals, which on the one hand could be considered weird experiments of artificial selection and not the result of true evolution in nature with its manifold ‘natural mutants’ [158,159], might be construed as misguided or a waste of time. However, on the other hand, domesticated animals do offer subjects of study that are potentially more accessible than wild species, and the possibilities of integrating evo–devo with other approaches such as population genetics, archaeology and genomics are seemingly endless. Even though domestication processes are like experiments in artificial selection, they have been underused in organismal biology studies of morphology and development, as well as in the investigation of physiological variables [160,161]. Moreover, the developmental and organismal approaches needed to understand domestication are tied to major concepts in evo–devo and evolutionary biology at large, (e.g. evolvability, modularity and developmental plasticity), and thus many testable hypotheses and intriguing opportunities remain.
